# Telerehabilitation as an innovative strategy for the management of anxiety and dyspnea in post‐COVID‐19: A scoping review

**DOI:** 10.1002/pmrj.13403

**Published:** 2025-05-27

**Authors:** Gustavo Elias Ferreira Neto, Guilherme Diniz Prudente, Henrique Lima de Oliveira, Khissya Beatryz Alves de Lima, Antônio da Silva Menezes Junior

**Affiliations:** ^1^ Department of Medicine Medical and Life School, Pontifical Catholic University of Goiás Goiás Brazil; ^2^ Internal Medicine Department Medicine School, Federal University of Goiás Goiânia Brazil

## Abstract

The COVID‐19 pandemic brought challenges for everyone, especially for patients with persistent sequelae, driving interest in telerehabilitation as an alternative treatment. Additional evidence may be useful to better assess its efficacy and applicability in managing post‐COVID‐19 symptoms. This study aimed to enhance the understanding of telerehabilitation in the post‐COVID‐19 context, facilitating its integration into clinical settings and patient management. Adhering to Preferred Reporting Items for Systematic Reviews and Meta‐Analyses Extension for Scoping Reviews guidelines, we reviewed PubMed, Excerpta Medica, and Scopus databases until January 2024. The study included primary and secondary research evaluating the benefits and drawbacks of telerehabilitation for patients with persistent COVID sequelae. This review analyzed 19 studies on telerehabilitation for long‐term COVID‐19 patients. Key findings included the comparison between hospital‐based and telerehabilitation and synchronous versus asynchronous telerehabilitation. The main sequelae addressed were dyspnea, quality of life, and anxiety. Limitations, particularly regarding costs, were also examined. Telerehabilitation provides psychological and social support, which is essential for managing post‐COVID‐19. Despite initial costs, long‐term benefits include reduced anxiety and improved quality of life. More long‐term research is needed to better understand the limitations and potential implications of telerehabilitation for integration into post‐COVID‐19 care to optimize outcomes and provide continuous support to patients and caregivers during recovery.

## INTRODUCTION

Persistent symptoms of COVID‐19 were initially known as postacute COVID or post‐COVID syndrome.^1^, ^2^, ^3^ It is characterized by persistent symptoms that can be classified into clusters. In this work we included articles that studied patients with postacute (between 6 weeks and 12 weeks) and long COVID symptoms, the latter term being characterized by the presence of symptoms for more than 12 weeks and the most frequent in the literature.^1^, ^3^ These patients experience ongoing issues, such as shortness of breath, postexertional malaise (PEM), fatigue, chest pain, anxiety, depression, stress, and significant changes in their quality of life.^1^, ^2^, ^3^, ^4^ A significant number of individuals continue to experience chronic and persistent symptoms even after testing negative for COVID‐19, affecting their daily activities and work with notable socioeconomic implications.^3^, ^4^

Rehabilitation, historically characterized by a multifaceted spectrum that includes physical training, health education, and psychological support, faces new challenges imposed by lifestyle changes and technological advancements.^5^ These limitations for rehabilitation include geographic, financial, and time‐related barriers, due to the travel required for access, the related cost, and the limited time availability‐factors that affect both patients and health professionals.^1^, ^3^, ^5^ In addition, the COVID‐19 pandemic highlighted the need to make health services viable remotely to control infections transmitted by droplets and aerosols, in addition to limiting unnecessary in‐person visits.^1^, ^5^ Telerehabilitation has emerged as a promising alternative for post‐COVID‐19 patients to reduce risks.^6^, ^7^ Use of new communication technologies allows for safe and monitored rehabilitation care to be provided at the patient's home or any other location in real time or asynchronously, thereby reducing barriers related to time, distance, cost, and risk.^1^, ^4^

Telerehabilitation presents a considerable advance in terms of safety and accessibility. Studies by Bernal‐Ultera et al.^8^ and Vieira et al.^9^ have shown that this approach is safe with an adverse event rate comparable to conventional treatment, mostly mild or moderate. Its application expands the reach of rehabilitation services to patients who have difficulty accessing health centers, opening new treatment opportunities for a wide range of patients.^10^, ^11^ However, it is essential to better understand the benefits of telerehabilitation and investigate its advantages as well as any associated difficulties and limitations.^12^

Physiatrists investigated the applicability and effectiveness of telerehabilitation during the pandemic,^13^ and more robust scientific evidence is still needed to support this practice in the post‐COVID‐19 landscape.^10^ The questions regarding the applicability of telerehabilitation are focused on the limitations in relation to face‐to‐face consultations, such as the lack of tactile feedback and a more detailed examination, in addition to the applicability of the treatment in practice, due to the absence of a professional nearby to perform the exercises.^4^ Moreover, telerehabilitation also faces challenges such as low technological familiarity, technical issues, and lack of physical contact.^14^ Therefore, this study aimed to expand the understanding of telerehabilitation in the post‐COVID‐19 context, seeking to simplify its practical integration into clinical and patient management, emphasizing the particular benefits of this approach while considering its related limitations.

## METHODOLOGY

### Study Type

This is a scoping literature review based on the stages proposed by the Preferred Reporting Items for Systematic Reviews and Meta‐Analysis Extension for Scoping Reviews.^15^ Electronic databases such as PubMed, Embase, and Scopus were searched until January 2024 using the combination of MeSH terms “Telerehabilitation,” “COVID,” AND “Physiotherapy.” Additionally, bibliographic references were manually checked for the relevant articles included in this review.

### Review Question

This review question was formulated using the PCC strategy. Problem: long COVID; Conceptual Framework: telerehabilitation; Context: post‐COVID‐19 pandemic. Thus, it was: “What are the advantages and disadvantages of telerehabilitation in managing clinical sequelae in post‐COVID‐19 patients?”

### Protocol and Registration

Our protocol was drafted using the Preferred Reporting Items for Systematic Reviews and Meta‐analysis Protocols Extension for Scoping Reviews, which was revised by the research team and members of our study group and was disseminated through our program's Twitter account and newsletter to solicit additional feedback. The final protocol was registered prospectively with the Open Science Framework on February 1, 2024 (DOI: 10.17605/OSF.IO/AHGCR).

### Eligibility Criteria

To be included in this review, papers needed to measure the benefits and disadvantages of telerehabilitation as a therapeutic means for patients with long COVID sequelae, developed in the conceptual framework. Peer‐reviewed journal papers were included if they were published in any period, written in English, and involving human participants. Quantitative, qualitative, and mixed‐method studies were included to consider different aspects of measuring telerehabilitation as a therapeutic means. Papers were excluded if they did not fit into the study's conceptual framework, were focused on other chronic conditions, and were based on secondary sources such as editorials, books, expert opinion articles, dissertations, theses, and abstracts.

### Information Sources and Search Strategy

To identify potentially relevant documents, the following bibliographic databases were searched up to February 2024: MEDLINE, EMBASE, PubMed, MEDLINE, and Scopus. Two experienced authors (G. E.F.N and A.S.M. Jr.) drafted the search strategies and further refined them through team discussion. The final search strategy for MEDLINE can be found in Additional file Data S1. The final search results were exported into EndNote, and duplicates were removed by a library technician. The electronic database search was supplemented by searching the RAYYAN website (https://www.cmpa-acpm.ca/en) and scanning relevant reviews.

To increase consistency among reviewers, all reviewers screened the same 50 publications, discussed the results, and amended the screening and data extraction manual before beginning screening for this review. Nine reviewers working in pairs sequentially evaluated the titles, abstracts, and then full text of all publications identified by our searches for potentially relevant publications. We resolved disagreements on study selection and data extraction by consensus and discussion with other reviewers if needed.

### Data Charting Process

Two reviewers jointly developed a data‐charting form to determine which variables to extract. The two reviewers independently charted the data, discussed the results, and continuously updated the data‐charting form in an iterative process.

### Data Items

We abstracted data on article characteristics (eg, country of origin, funder), engagement characteristics, and contextual factors (eg, author/year of the study, country where the study was developed, title [identification], type of study, study objective, study methodology, and study conclusion), barriers and facilitators to engagement, and results of any formal assessment of engagement (eg, attitudes, beliefs, knowledge, benefits, unintended consequences).

### Risk of Bias or Quality Assessment

According to the manual published by the Joanna Briggs Institute, as a scoping review was conducted to identify knowledge gaps, there was no risk of bias or quality assessment.

### Data Synthesis

A qualitative synthesis of the data from the selected studies is provided. It describes the benefits of telerehabilitation in addressing long COVID sequelae as proposed in each study, evaluates the quality improvement of the sequelae patients experienced, or discusses the challenges telerehabilitation faces for implementation in the current context. A descriptive Table 1 summarizes this information.

**TABLE 1:** Description of selected studies.

Author/Year	Country	Title	Type of study	Methods	Main findings
Espinoza‐Bravo et al., 2023 ^4^	Chile	Effectiveness of Functional or Aerobic Exercise Combined with Breathing Techniques in Telerehabilitation for Patients with Long COVID: A Randomized Controlled Trial	Randomized controlled trial	The study used a randomized controlled trial methodology to compare the short‐term clinical effects of functional exercise and aerobic exercise, combined with breathing techniques, on improving symptoms of long COVID‐19.	Both telerehabilitation modalities, functional and aerobic exercise, were effective in improving stress symptoms and quality of life in patients with long COVID‐19 durations, with functional exercises showing more promising results for fatigue and functional performance.
Mavronasou et al., 2023 ^16^	Greece	Remote Administration of the Short Physical Performance Battery, the 1‐Minute Sit To Stand, and the Chester Step Test in Post‐COVID‐19 Patients after Hospitalization: Establishing Inter‐Reliability and Agreement with the Face‐to‐Face Assessment	Observational study	Twenty‐five patients who were post‐COVID‐19 underwent random assessments using the Short Physical Performance Battery, 1‐min Sit to Stand Test, and Chester Step Test through remote evaluation using a software platform at home and face‐to‐face assessment at a rehabilitation center, with a 24‐ to 72‐h interval.	Remote assessments of the SPPB, 1‐MSTS, and CST showed moderate to excellent interrater reliability in patients post‐COVID‐19 after hospitalization, providing valuable insights for implementing remote evaluations in this population.
Plaza et al., 2022 ^17^	Spain	Telematics Program of Breathing Exercises and Mindfulness for Patients with Post‐COVID‐19	Quasi‐experimental study	The study employed a quasi‐experimental design involving patients post‐COVID‐19, using remote respiratory physiotherapy and a mindfulness program.	The findings suggest that remote respiratory physiotherapy and mindfulness, as part of a multidisciplinary approach, effectively reduce dyspnea and anxiety while enhancing the quality of life in patients post‐COVID‐19 during confinement.
Vieira et al., 2022 ^9^	Brazil	Telerehabilitation Improves Physical Function and Reduces Dyspnea in People with COVID‐19 and Post‐COVID‐19 Conditions: A Systematic Review.	Systematic review	The methodology involved a systematic review of randomized controlled trials. Database searches were conducted, resulting in the inclusion of six studies with a total of 323 participants.	Telerehabilitation may improve functional capacity, dyspnea, performance, and physical quality of life for individuals with COVID‐19 and post‐COVID‐19 conditions. Additionally, the study found that telerehabilitation does not substantially increase adverse events.
Reis et al., 2022 ^1^	Portugal	Telerehabilitation in the Transitional Care of Patients with Sequelae Associated with COVID‐19: Perception of Portuguese Nurses	Qualitative research study	The study employed an online focus group method with eight nurses specialized in rehabilitation nursing. The responses to semistructured interviews were subjected to content analysis, and qualitative data analysis software (WebQDA®) was used to organize and analyze the findings.	The study identified crucial aspects to consider in planning and implementing telerehabilitation interventions for transitional care in individuals with long COVID‐19 after hospitalization. The findings contribute significantly to the reorganization of transitional care and offer insights into the advantages and opportunities associated with telerehabilitation.
Valverde‐ Martínez et al., 2023 ^3^	Spain	Telerehabilitation, A Viable Option in Patients with Persistent Post‐COVID Syndrome: A Systematic Review	Systematic review	The study conducted a systematic review, analyzing six articles and assessing 140 patients with post‐COVID‐19 syndrome.	The study's conclusion suggests that telerehabilitation could be an effective tool for treating persistent symptoms in individuals with post‐COVID‐19 syndrome. The interventions included various exercises and innovative techniques, which improved physical performance and quality of life in the analyzed patients.
Estebanez‐Pérez et al., 2023 ^10^	Spain	Effectiveness of Digital Physiotherapy Practice Compared to Usual Care in Long COVID Patients: A Systematic Review	Systematic review	A systematic review was conducted, using information available on four databases until November 2022. The search terms included MeSH terms related to post‐COVID syndrome, telerehabilitation, physiotherapy, rehabilitation, virtual, and home care. The study included six articles detailing the symptomatology, assessment, treatment, and monitoring of 140 patients. The variables measured included dyspnea, fatigue, physical performance, and quality of life.	The study's conclusion highlights that telerehabilitation can effectively treat persistent symptoms after COVID‐19 infection. The results indicate improvements in physical performance and quality of life among patients, demonstrating the effectiveness of the telerehabilitation interventions addressed in the analyzed studies.
M De‐La‐Plaza‐San‐Frutos et al., 2021 ^18^	Spain	Telemedicine in Pulmonary Rehabilitation – Benefits of a Telerehabilitation Program in Post‐COVID‐19 Patients: A Controlled Quasi‐Experimental Study	Quasi‐experimental study	A quasi‐experimental study on patients who were post‐COVID‐19 assessed the impact of breathing exercises and mindfulness, remotely supervised by a respiratory physiotherapist. Dyspnea on exertion, quality of life, and anxiety levels were measured before and after the rehabilitation program.	The study found that a multidisciplinary approach, including respiratory rehabilitation and mindfulness, effectively reduced dyspnea and anxiety and improved quality of life in patients post‐COVID‐19, highlighting the limited research in this area.
Bernal‐Utrera, C.et al., 2022 ^8^	Spain	Therapeutic Exercise Interventions through Telerehabilitation in Patients with Post‐COVID‐19 Symptoms: A Systematic Review	Systematic review	Research across four databases identified five studies, leading to categorization into two subgroups based on patient symptoms post‐COVID‐19: post discharge and long term (over 2 mo) symptoms.	Telerehabilitation, incorporating aerobic, respiratory, and strength exercises, effectively improved cardiovascular and cardiorespiratory outcomes for patients with short‐ and long‐term post‐COVID‐19 sequelae.
Rodriguez‐Blanco, C et al., 2023 ^19^	Spain	A 14‐Day Therapeutic Exercise Telerehabilitation Protocol of Physiotherapy Is Effective in Non‐Hospitalized Post‐COVID‐19 Conditions: A Randomized Controlled Trial	Randomized controlled trial	A randomized controlled study analyzed data from 48 patients through the BS, 30STST, MD12, VAFS, and 6MWT assessments.	The results demonstrated that the intervention group significantly outperformed the control group in achieving positive outcomes.
Estebanez‐Pérez, M.‐J. et al., 2022 ^10^	Spain	The Effectiveness of a Four‐Week Digital Physiotherapy Intervention to Improve Functional Capacity and Adherence to Intervention in Patients with Long COVID‐19	Quasi‐experimental clinical trial	A study of 32 participants evaluated functional capacity and treatment adherence before and after a 4‐wk digital physiotherapy intervention, noting significant improvements and high adherence rates.	The researcher considered the intervention feasible, safe, and aligned with the objectives. However, they noted the need for further randomized clinical trials and studies with larger sample sizes to make generalizable conclusions.
Calvache‐Mateo, A. et al., 2023 ^20^	Spain	Efficacy and Safety of Respiratory Telerehabilitation in Patients with Long COVID‐19: A Systematic Review and Meta‐Analysis	Systematic review and meta‐analysis	A systematic review and meta‐analysis on the effects of respiratory telerehabilitation in patients with long COVID‐19 compared to controls showed significant improvements in quality of life, dyspnea, respiratory muscle strength, functional capacity, and lower limb strength, but not in lung function or anxiety and depression, with no significant difference in adverse effects.	The findings indicate that these interventions can enhance life quality, alleviate dyspnea, and boost respiratory and lower limb muscle strength and functional capacity in patients with long COVID‐19.
Calvo‐Paniagua J. et al., 2022 ^21^	Spain	A Telehealth Primary Care Rehabilitation Program Improves Self‐Perceived Exertion in COVID‐19 Survivors Experiencing Post‐COVID Fatigue and Dyspnea: A Quasi‐Experimental Study	Quasi‐experimental study	Sixty‐eight post‐COVID fatigue and dyspnea patients at four primary health care centers in Madrid joined an 18‐session telerehabilitation program involving patient education, physical activity, airway clearing, and breathing exercises (three sessions/wk).	Telerehabilitation programs offer an effective strategy for alleviating post‐COVID fatigue and dyspnea in survivors, potentially reducing the economic burden of acute COVID‐19 by accommodating more patients and freeing up intensive care unit beds for those with severe disease.
Kortianou E. et al., 2022 ^22^	Greece	Application of a Home‐Based Exercise Program Combined with Telerehabilitation in Previously Hospitalized Patients with COVID‐19: A Feasibility, Single‐Cohort Interventional Study	Single‐cohort interventional study	The study used a pre‐ and post‐intervention design in two phases. Initially, patients received instructions on using a COVID‐19‐specific e‐book during four telehealth sessions. Subsequently, a 2‐mo home‐based program included self‐practice exercises and supervised telerehabilitation sessions lasting 1 hr every 10 d.	Telerehabilitation may be feasible and improve the physical and psychological status of patients with COVID‐19 after hospital discharge.
Mheiri A. et al., 2023 ^23^	United Arab Emirates	Effects of Six Weeks of Supervised Telerehabilitation on Pulmonary Function, Functional Capacity and Dyspnoea among Individuals with Long COVID	Single‐group pretest‐posttest quasiexperimental design	A 6‐wk telerehabilitation program was implemented for patients aged 18–75 with long COVID, using a single‐group pretest‐posttest quasiexperimental design. Pre‐ and postassessments included the 6MWT, PFTs, mMRC scale for dyspnea, and the GPAQ. The study also monitored technical issues and session adherence.	This approach significantly improved functional capacity and pulmonary function, reduced dyspnea, and enhanced physical activity levels in individuals with long COVID. The study's findings underscore the feasibility and effectiveness of telerehabilitation programs tailored to this patient group.
da Silva et al., 2023 ^24^	Brazil	Effects of a Cardiopulmonary Telerehabilitation Using Functional Exercises in Individuals after COVID‐19 Hospital Discharge: A Randomized Controlled Trial	Randomized controlled trial	This blinded, randomized, controlled clinical trial included 67 adult individuals after COVID‐19 hospital discharge. Participants were randomized into the groups of telerehabilitation (TG; *n* = 33) and control (CG; *n* = 34). The TG underwent an individualized exercise program (functional and accessible exercises) supervised by a physical therapist (videoconference), and the CG received guidance on general care and self‐monitoring of vital signs (videoconference)	Cardiopulmonary telerehabilitation using functional exercises improved the exercise and functional capacity assessed using 6MST, 30CST, and 2MSWT and the quality of life of individuals after COVID‐19 hospital discharge.
T. del Corral et al., 2022 ^25^	Spain	Home‐Based Respiratory Muscle Training on Quality of Life and Exercise Tolerance in Long‐Term Post‐COVID‐19: Randomized controlled trial	Randomized controlled trial	Eighty‐eight individuals with long‐term symptoms of fatigue and dyspnea after COVID‐19 diagnosis were randomly (1:1 ratio) assigned to IMT, IMT_sham_, RMT, or RMT_sham_ groups for an 8‐wk intervention (40 min/d, six times/wk).	Only an 8‐wk supervised home‐based RMT program was effective in improving quality of life, but not exercise tolerance, in individuals with long‐term post‐COVID‐19 symptoms.
Furtado P. et al., 2023 ^26^	Brazil	The Effect of Telerehabilitation on Physical Fitness and Depression/Anxiety in Post‐COVID‐19 Patients: A Randomized Controlled Trial	Randomized controlled trial	Thirty‐two individuals who recovered from COVID‐19 (48.20 ± 12.82 y) were allocated into either a telerehabilitation (TG *n* = 16) or control (CG *n* = 16) group. Physical fitness, handgrip strength, depression, and anxiety levels were assessed before and after an 8‐wk intervention.	Eight wks of functional telerehabilitation training is a viable and efficient way to rehabilitate patients affected by COVID‐19, as it improves physical conditioning and mental health.
Dalbosco‐Salas M. et al., 2021 ^27^	Chile	Effectiveness of a Primary Care Telerehabilitation Program for Post‐COVID‐19 Patients: A Feasibility Study	Observational prospective study	An observational, prospective study was conducted in seven primary care centers in Chile. The telerehabilitation program consisted of 24 sessions of supervised home‐based exercise training. The efficacy was measured by the 1‐min STST, the 36‐item Short Form Health Survey, fatigue, and dyspnea symptoms before and after intervention.	A telerehabilitation program applied in primary health care is feasible and effective in improving physical capacity, quality of life, and symptoms in adult survivors of COVID‐19.

Abbreviations: 1‐min STST, 1‐minute sit‐to‐stand test; 1‐MSTS, 1‐minute Sit to Stand Test; 2MSWT, 2‐minute stationary walk test; 6MST, 6‐minute step test; 6MWT, the six‐minute walk test; 30CST, 30‐second chair stand test; 30STST, 30‐Second Sit‐to‐Stand Test; BS, Borg Scale; CST, Chester Step Test; GPAQ, Global Physical Activity Questionnaire; IMT, inspiratory muscle training; MD12, Multidimensional Dyspnea‐12; MeSH, Medical Subject Headings; mMRC, modified Medical Research Council Dyspnea scale; PFTs, pulmonary function tests; RMT, inspiratory/expiratory muscle training; SPPB, Short Physical Performance Battery; VAFS, Visual Analog Fatigue.

## RESULTS AND DISCUSSION

**FIGURE 1:**
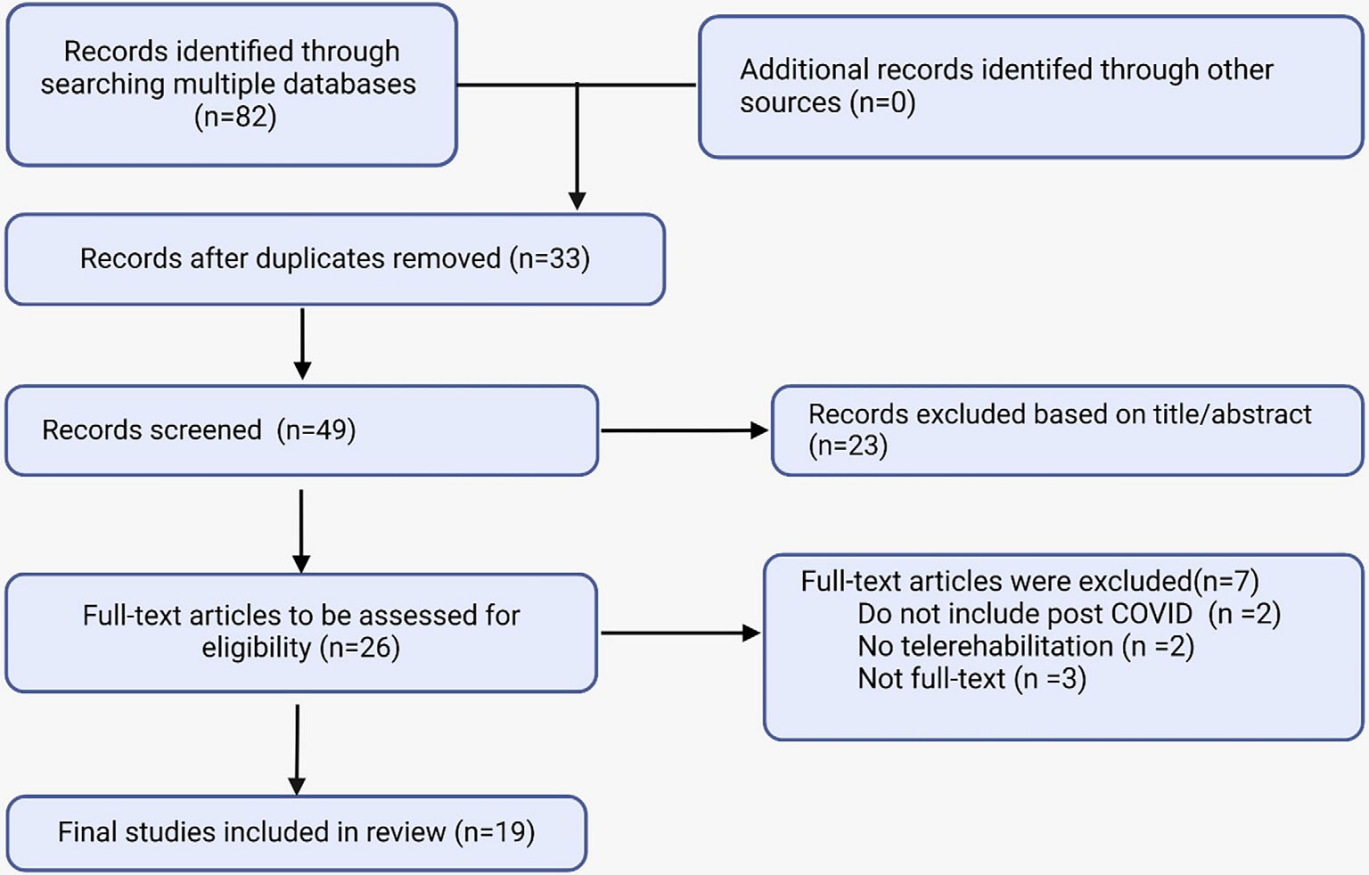
PRISMA flow chart of the reviewed articles. PRISMA, Preferred Reporting Items for Systematic Reviews and Meta‐Analyses.

**FIGURE 2:**
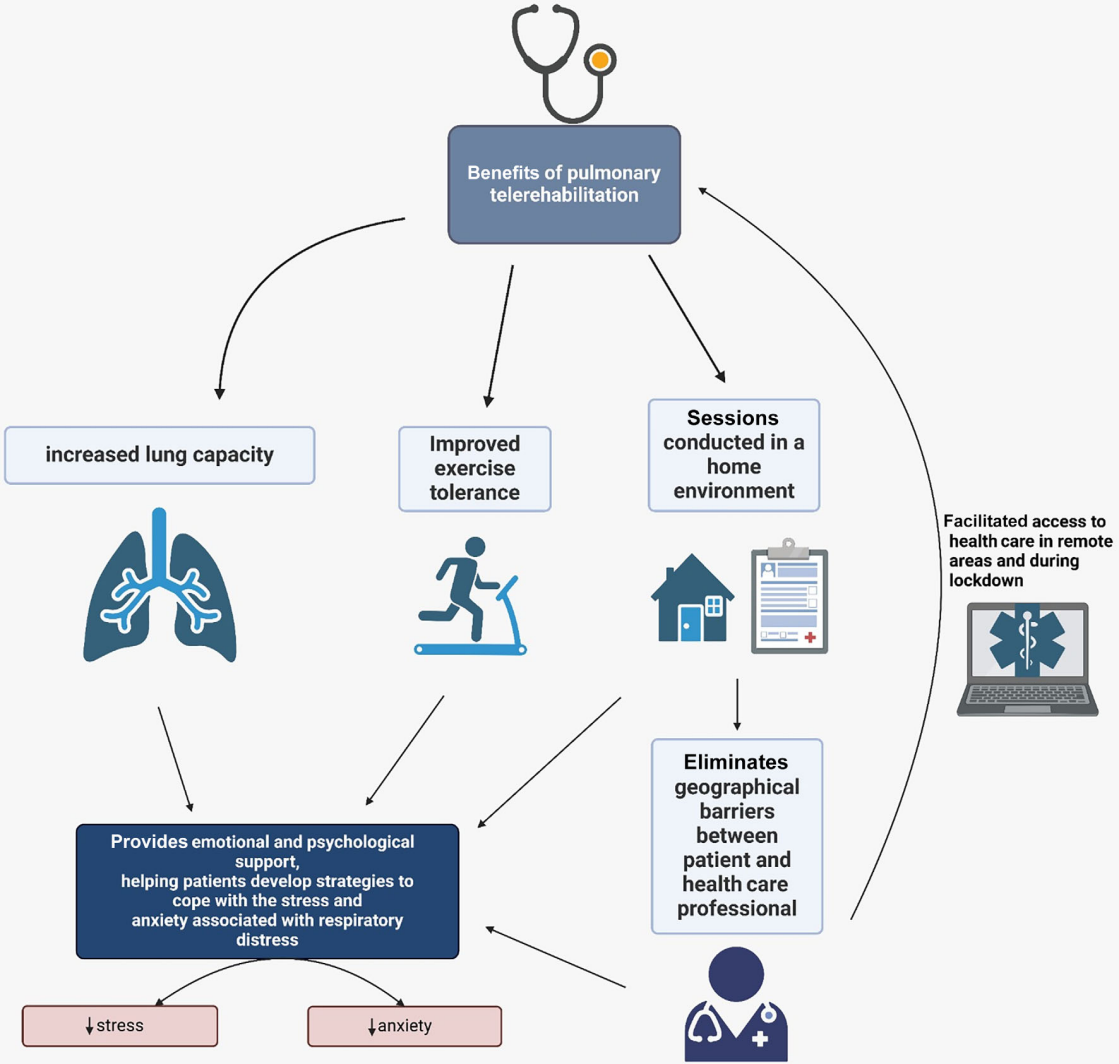
Benefits of pulmonary telerehabilitation on physical capacity, emotional support, and remote access to health.

**FIGURE 3:**
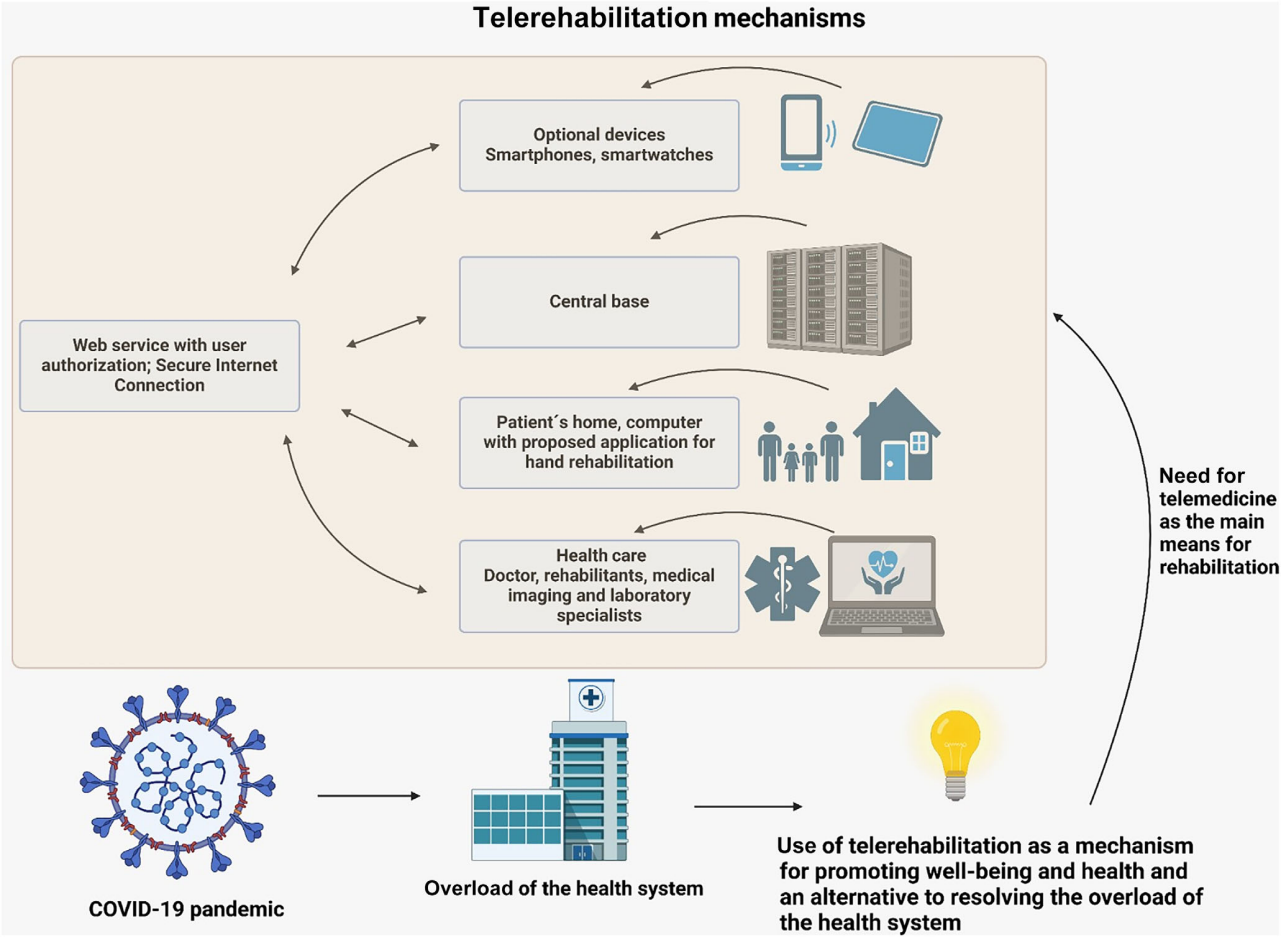
Mechanisms of telerehabilitation and the impact on optimizing the health system during COVID‐19.

A total of 82 articles were selected; 33 duplicates and 30 papers not related to the selected group for research, not having the full text available, or not addressing telerehabilitation as the main discussion point were excluded. Therefore, 19 articles were selected for the final analysis, as shown in Figure 1. The primary data of each study are presented in Table S1. The analysis categories were (1) hospital‐based rehabilitation versus telerehabilitation; (2) synchronous versus asynchronous telerehabilitation; (3) dyspnea; (4) quality of life; (5) anxiety level; and (6) limitations of telerehabilitation (Figures 2 and 3).

The analysis categories were created by carefully reading the articles and observing the main discussion points. Six main topics were raised surrounding the central idea of each article, making them relevant to provide the necessary theoretical basis for discussing the real benefits of cardiac telerehabilitation in the post‐COVID‐19 context.

### Hospital‐Based Rehabilitation versus Telerehabilitation

The pandemic inevitably burdened health care systems, resulting in high hospitalization and global mortality rates.^9^ Consequently, telerehabilitation emerged as an emergency solution to overcome the difficulties faced in conventional rehabilitation, providing an approach that aims to reduce risk and decrease contagion.^11^, ^15^

Indeed, the COVID‐19 pandemic offered an opportunity to expand telerehabilitation globally, not only for patients with COVID‐19 but also those with chronic conditions.^28^, ^29^ Studies confirm that this modality provides benefits comparable to in‐person rehabilitation while minimizing barriers related to distance, time, and costs.^8^, ^10^ Randomized studies highlight the effectiveness of telerehabilitation for post‐COVID‐19 patients who were hospitalized, revealing potential therapeutic benefits with increased lung function.^30^, ^31^ Both results indicate that an online breathing program can improve both dyspnea and quality of life, representing a potential complementary therapy for rehabilitating individuals with persistent symptoms of COVID‐19.^29^, ^30^

### Synchronous versus Asynchronous Telerehabilitation

The use of technology within telerehabilitation, such as videoconferences, has been adopted worldwide in various formats. These include synchronous sessions, where patients and therapists interact in real time through devices; asynchronous, where the intervention is remotely monitored without the immediate presence of a therapist during the session; or a combination of both.^32^, ^33^ Telerehabilitation can be facilitated by various technologies such as virtual assistants and communication platforms integrated with enterprise resource planning systems.^33^ In particular, mobile applications have demonstrated the capacity to reduce demand in hospitals, offer access to information, and monitor possible symptoms and individuals' mental health status.^34^ Espinoza‐Bravo et al. evaluated post‐COVID patients for 8 weeks using the mobile app Fisiotrack, demonstrating patient adherence rates >89%.^4^ Other studies also analyzed the use of mobile apps, such as the study by Li et al., which evaluated an unsupervised 6‐week home exercise program performed through a smartphone app called RehabApp and monitored by a heart rate telemetry device worn on the chest.^35^ Adherence in this study was considered satisfactory.

Among the techniques proposed for telerehabilitation, strategies for breathing control and chest expansion have been proposed as therapeutic alternatives for COVID‐19 patients and have been incorporated into physiotherapy protocols for prolonged COVID‐19 cases.^35^ These protocols include aerobic activities and exercises to strengthen large muscle groups, demonstrating benefits in terms of dyspnea, exercise capacity, pulmonary function measures, and quality of life.^36^, ^37^ Espinoza‐Bravo et al. demonstrated that functional exercises improve fatigue and functional performance.^4^

Considering the ways in which rehabilitation is used, the use of non‐face‐to‐face assessment was also raised.^16^, ^30^, ^38^, ^39^ The study conducted by Mavronasou et al. evaluated the Short Physical Performance Battery (SPPB), 1‐minute Sit‐to‐Stand Test (1‐MSTS), and Chester Step Test (CST) through remote assessment of patients post‐COVID‐19 after hospitalization.^16^ These assessment techniques were also found in other studies and were validated with moderate‐to‐excellent reliability in these patients.^30^, ^38^, ^39^ The SPPB comprises a series of brief physical tests to evaluate an individual's functional capacity and physical performance, particularly in older adults, including balance, gait speed, and lower‐limb strength assessments. The 1‐MSTS, in turn, aims to assess the strength and endurance of the lower limbs by measuring how many times a person can stand up and sit in a chair within 1 minute.^16^ Finally, the CST is a cardiovascular fitness assessment focused on an individual's aerobic capacity. During the test, heart rate was monitored before, during, and after the activity, allowing the determination of cardiovascular capacity and the efficiency of the cardiorespiratory system in supplying oxygen to the muscles during physical exercise.^16^

Among other tests administered to patients, the 6‐minute walking distance test was used to assess functional capacity and exercise tolerance, with the distance covered by the patient during the 6 minutes being recorded. This provides an objective measure to monitor progression of a patient's condition over time and evaluate treatment effectiveness.^35^, ^40^

Other patient monitoring and control mechanisms can also be used, such as the London Chest Activity of Daily Living scale, which is a specific dyspnea assessment instrument developed and validated for individuals with chronic obstructive pulmonary disease.^41^ It is associated with different objective assessment instruments for functional capacity and physical activity in daily life and demonstrates responsiveness to pulmonary rehabilitation programs. This scale was validated by Silva et al. in patients post‐COVID during telerehabilitation, making the analysis more accessible to patients undergoing this type of remote treatment.^42^

From a synchronous monitoring perspective, Rodriguez‐Blanco et al. used two methods to assess dyspnea and perceived exertion in patients post‐COVID: Multidimensional Dyspnea‐12 (MD12) and the modified Borg Scale of Perceived Exertion (BS).^19^ The BS sets standards for adjusting exercise intensity or workload and predicting and dictating various effects of sports and medical rehabilitation activities. Both methods were categorized through forms evaluated by a multidisciplinary team and patients via video calls at 14‐day intervals, with consultations on the first and 14th days supervised by a physiotherapist, reinforcing the exercise plan through telematic control in video conferences with each participant.^19^ Thus, the results were favorable for the MD12 and BS assessment measures.^19^ Additionally, patients who participated in a similar telerehabilitation program reported positive results, confirming the reliability of the measures for categorizing symptomatic improvement presented by patients.^38^

Tanhan et al. used four scales to measure the quality of asynchronous and synchronous telerehabilitation programs: the Incremental Shuttle Walk Test, Short Physical Performance Battery (SPPB), Health‐Related Quality of Life, and Hospital Anxiety and Depression Scale.^43^ According to Tanhan et al., both asynchronous and synchronous telerehabilitation effectively treat clinical and functional characteristics when properly planned and implemented.^43^

### The Role of Telerehabilitation in Treating Respiratory Difficulty

In patients post‐COVID‐19, telerehabilitation offers a promising approach to improve lung function and reduce respiratory symptoms, representing a safe and effective alternative to traditional rehabilitation.^10^, ^17^ Personalized exercise programs monitored by medical professionals through online platforms promote respiratory recovery and reduce shortness of breath.^17^, ^19^ Estebanez‐Pérez et al. found considerable data on the improvement of dyspnea in patients with long COVID, accounting for an improvement in modified Medical Research Council Dyspnea (mMRC) scale score of 90.4% of patients.^10^ Mheiri et al. demonstrated a variation in mMRC score of 47.8% before and after telerehabilitation, in favor of improving dyspnea.^23^

Significant improvements in patients undergoing telerehabilitation include increased lung capacity, improved exercise tolerance, and reduced shortness of breath.^4^, ^23^ Rehabilitation sessions can be conducted in a home environment, eliminating geographic barriers, and facilitating access to health care, especially in remote areas and during social distancing.^17^, ^27^

Shortness of breath after COVID‐19 not only affects patients' physical capacity but also has a significant psychological impact.^36^, ^37^ The sensation of shortness of breath can cause anxiety and fear, triggering a cycle of symptoms that are further exacerbated by health problems.^22^, ^27^ In this sense, telerehabilitation can address the physical aspects of shortness of breath, and provide emotional and psychological support, helping patients develop strategies to cope with stress and anxiety associated with respiratory difficulty.^24^, ^44^

Health care professionals can tailor interventions based on patient progress through remote monitoring and continuous adjustment of training programs to ensure effective and safe treatment.^10^, ^45^ This personalized approach optimizes treatment outcomes and makes patients feel supported and motivated as they observe progress over time, improving adherence to treatment.^16^, ^39^, ^45^

In this context, telerehabilitation has proven to be a valuable tool for treating respiratory difficulties in patients post‐COVID‐19.^9^, ^10^, ^21^, ^30^ This treatment is essential in improving quality of life and promoting respiratory recovery in survivors of COVID‐19, providing an accessible, personalized, and safe intervention.^9^

### Quality of Life

The COVID‐19 pandemic not only poses challenges to individuals' physical health but also has a significant impact on their quality of life.^8^, ^20^ Recovery from an infectious disease can involve various emotional, social, and physical challenges that can affect a patient's overall health status.^8^ Telerehabilitation has proven to be a promising tool for improving patients' quality of life.^20^

Telerehabilitation has positively impacted the quality of life of individuals facing long COVID syndrome in a statistically significant manner (*p* <.05).^3^, ^25^, ^26^ Regarding physical performance, patients reported improvements in their physical endurance and ability to perform daily activities.^25^ Socially, telerehabilitation has allowed patients to connect better with their support networks, even remotely, reducing feelings of isolation and loneliness.^46^ Participating remotely in rehabilitation sessions also eliminates geographic barriers, enabling patients in remote areas or with limited mobility to access necessary care.^46^

The COVID‐19 pandemic affected those living with the disease and their families and caregivers.^47^, ^48^ The emotional stress and anxiety associated with caring for a loved one with COVID‐19 can be overwhelming, increasing the need for supportive interventions for both patients and those around them.^47^ In addition to providing direct support to patients, telerehabilitation can be essential in providing emotional support and guidance to caregivers, reducing the stress and fatigue associated with caring for sick relatives.^47^, ^49^, ^50^

These studies highlight the significant role of telerehabilitation in promoting overall well‐being and life satisfaction in patients post‐COVID‐19.^51^, ^52^ Telerehabilitation has proven to be an essential tool in managing the effects of this disease, providing a comprehensive approach that addresses the physical, emotional, and social aspects of recovery.^51^

### Anxiety

Anxiety is a common concern among patients with COVID‐19 and is often exacerbated by the uncertainty surrounding the disease and the emotional stress associated with health care experiences.^12^, ^43^ The effect of telerehabilitation on reducing anxiety in patients after COVID‐19 has been investigated, providing valuable insights into the potential of remote interventions to offer psychological support and coping strategies.^26^

Telerehabilitation sessions provide the opportunity to learn relaxation techniques, breathing, and mindfulness, which have proven effective in reducing stress and promoting mental health.^25^ Teleintervention offers a safe and accessible platform for patients to express concerns and receive advice from qualified health care professionals.^50^ Therefore, telerehabilitation has had a positive effect on reducing anxiety in patients post‐COVID‐19.^9^, ^20^, ^50^, ^51^

Telerehabilitation provides a flexible and adaptable approach that meets individual patient needs and ensures continuous support after discharge.^12^, ^45^ This continuity of care is critical because anxiety can persist throughout the recovery process, and the ability to remotely access support resources, especially when facing unexpected challenges or moments of greater emotional vulnerability, can be reassuring for patients.^12^, ^53^, ^54^

Telerehabilitation can play a crucial role in preventing long‐term mental health complications related to COVID‐19.^9^, ^26^ Research shows that patients with high levels of anxiety during recovery may be at a greater risk of developing mental health disorders, such as depression and posttraumatic stress disorder, even after the disease has been resolved.^54^, ^55^ Therefore, addressing fear early through remote intervention can reduce these risks and promote a more complete and resilient recovery.^55^, ^56^

By integrating telerehabilitation as part of post‐COVID‐19 care protocols, health care professionals can provide a more humane approach that addresses not only patients' physical needs but also their emotional and psychological needs.^57^ Thus, the significant role of telerehabilitation in improving mental health and anxiety and reducing stress in patients post‐COVID‐19 has been highlighted.^58^ Estebanez‐Pérez et al. stated that telerehabilitation was not significant in reducing anxiety levels; however, they reinforced that it helps in maintaining mental health.^10^

### Costs and Limitations of Telerehabilitation

Introducing telerehabilitation in the post‐COVID‐19 context raises important questions about the costs associated with this treatment approach.^59^ A comprehensive analysis should consider the investment in technology and infrastructure needed to provide remote services and the clinical and economic benefits that telerehabilitation can offer.^13^ Current studies, such as those by Frutos et al., have investigated the costs associated with telerehabilitation compared to traditional rehabilitation approaches.^18^ Although implementing telerehabilitation may have significant initial costs due to equipment acquisition and medical professionals' training, the long‐term clinical and economic benefits can offset these investments.^60^

However, evaluating the long‐term economic feasibility of telerehabilitation requires considering financing policies and payment models.^58^, ^61^ Appropriate reimbursement strategies are essential to ensure this care model's economic sustainability and promote widespread acceptance in the health care system.^61^, ^62^ Therefore, although telerehabilitation has initial costs, the potential clinical and economic benefits justify considering this innovative therapeutic approach in treating post‐COVID‐19 sequelae.^63^

Telerehabilitation lacks widely accepted, evidence‐based guidelines to ensure standardized and effective practice, which limits the confidence of professionals and patients in its efficacy and safety. In addition to the lack of clear regulation, privacy and confidentiality challenges remain a concern.^15^

Telerehabilitation offers a reduced experience when compared to in‐person care, which can negatively affect the quality of care, either due to lack of physical proximity and touch, or because communication needs to be mediated by devices, creating a less welcoming relationship between physiatrist and patient.^12^ In addition, many health professionals still do not have adequate training to perform rehabilitation remotely, which can affect the quality of care and patient satisfaction.^12^, ^15^

This review focused on analyzing symptoms of anxiety and dyspnea, but PEM was not addressed, even though it is a prevalent symptom in postacute COVID, as no articles were found that related telerehabilitation and PEM. The other phenotypes presented after the acute COVID condition could not be analyzed due to data limitations, and for this reason it is not possible to extrapolate to the phenotypes not used in this study. The other phenotypes of symptoms after the acute phase of COVID were considered, but there are still limitations in research aimed at correlating them with telerehabilitation. Furthermore, the studies do not explicitly address patient phenotypes or related processes, such as the etiology of dyspnea, limiting the analysis of the effectiveness of telerehabilitation in managing patients with persistent COVID symptoms.

### Limitations

The results of this research highlight a scarcity of articles directly related to telerehabilitaton in patients post‐COVID‐19. Despite the exhaustive search for articles, only a few are directly connected to the role of telerehabilitation and its benefits throughout the long COVID phase.

## CONCLUSION

In conclusion, the studies discussed highlight the significant role of telerehabilitation in managing post‐COVID‐19 impacts. This treatment aims for the patient's physical recovery, provides psychological and social support, reduces anxiety, improves quality of life, and adopts a flexible and adaptable approach to individual needs. Despite the initial costs, the long‐term clinical, economic, and psychological benefits support the widespread adoption of telerehabilitation as an integral part of post‐COVID‐19 care protocols. However, there are still some limitations in research studies on this topic, both in availability of quantitative data, and in better classification and delineation of symptoms. More research is needed to better identify the appropriate target population for telerehabilitation to avoid implementation for those patients who may not benefit. This promising approach optimizes treatment outcomes and provides continuous support to patients and caregivers during COVID‐19 recovery.

## DISCLOSURE

There are no conflicts of interest, financial or otherwise.

## Supporting information

Table S1

## Data Availability

The article's data will be shared by the corresponding author (A.M.) at a reasonable request.

## References

[1] Reis N , Dias MJC , Sousa L , et al. Telerehabilitation in the transitional care of patients with sequelae associated with COVID‐19: perception of Portuguese nurses. Int J Environ Res Public Health. 2022;19(24):17096. 36554975 10.3390/ijerph192417096 PMC9779261

[2] Nicholls SJ , Nelson M , Astley C , et al. Optimising secondary prevention and cardiac rehabilitation for atherosclerotic cardiovascular disease during the COVID‐19 pandemic: a position statement from the Cardiac Society of Australia and New Zealand (CSANZ). Heart Lung Circ. 2020;29(7):104. 10.1016/j.hlc.2020.04.007 PMC7192068 32473781

[3] Valverde‐Martínez MÁ , López‐Liria R , Martínez‐Cal J , Benzo‐Iglesias MJ , Torres‐Álamo L , Rocamora‐Pérez P . Telerehabilitation: a viable option in patients with persistent post‐COVID syndrome: a systematic review. Healthcare. 2023;11(2):187. 36673555 10.3390/healthcare11020187 PMC9859291

[4] Espinoza‐Bravo C , Arnal‐Gómez A , Martínez‐Arnau FM , et al. Effectiveness of functional or aerobic exercise combined with breathing techniques in telerehabilitation for patients with long COVID: a randomized controlled trial. Phys Ther. 2023;103(11):pzad118. 37658773 10.1093/ptj/pzad118

[5] de Castro RRT . Coronavirus disease (COVID‐19) pandemic: an opportunity window to implement home‐based cardiac rehabilitation. Int J Cardiovasc Sci. 2020;33:282‐283. doi:10.36660/ijcs.20200062

[6] Babu AS , Arena R , Ozemek C , Lavie CJ . COVID‐19: a time for alternate models in cardiac rehabilitation to take Centre stage. Can J Cardiol. 2020;36(6):792‐794. 32344000 10.1016/j.cjca.2020.04.023 PMC7195273

[7] Grace SL , Taylor RS , Gaalema DE , Redfern J , Kotseva K , Ghisi G . Cardiac rehabilitation: a global perspective on where we have come and where we must go. JACC Adv. 2023;2(5):100412. 38938991 10.1016/j.jacadv.2023.100412 PMC11198422

[8] Bernal‐Utrera C , Montero‐Almagro G , Anarte‐Lazo E , Gonzalez‐Gerez JJ , Rodriguez‐Blanco C , Saavedra‐Hernandez M . Therapeutic exercise interventions through telerehabilitation in patients with post COVID‐19 symptoms: a systematic review. J Clin Med. 2022;11(24):7521. 36556137 10.3390/jcm11247521 PMC9785416

[9] Vieira AGDS , Pinto ACPN , Garcia BMSP , Eid RAC , Mól CG , Nawa RK . Telerehabilitation improves physical function and reduces dyspnoea in people with COVID‐19 and post‐COVID‐19 conditions: a systematic review. J Physiother. 2022;68(2):90‐98. 35414491 10.1016/j.jphys.2022.03.011 PMC8994568

[10] Estebanez‐Pérez MJ , Martín‐Valero R , Vinolo‐Gil MJ , Pastora‐Bernal JM . Effectiveness of digital physiotherapy practice compared to usual Care in Long COVID patients: a systematic review. Healthcare. 2023;11(13):1970. 37444803 10.3390/healthcare11131970 PMC10340626

[11] Rogante M , Grigioni M , Cordella D , Giacomozzi C . Ten years of telerehabilitation: a literature overview of technologies and clinical applications. NeuroRehabilitation. 2010;27(4):287‐304. 21160118 10.3233/NRE-2010-0612

[12] Cordani C , Young VM , Arienti C , et al. Cognitive impairment, anxiety, and depression: a map of Cochrane evidence relevant to rehabilitation for people with post COVID‐19 condition. Eur J Phys Rehabil Med. 2022;58(6):880‐887. 36534008 10.23736/S1973-9087.22.07813-3 PMC10153550

[13] Leochico CFD , Rey‐Matias BMV , Rey‐Matias RR . Telerehabilitation perceptions and experiences of physiatrists in a lower‐middle‐income country during the COVID‐19 pandemic. PM&R. 2022;14(2):210‐216. 34585855 10.1002/pmrj.12715 PMC8661588

[14] Chuang HJ , Lin CW , Hsiao MY , Wang TG , Liang HW . Long COVID and rehabilitation. J Formos Med Assoc. 2024;123–9:S61‐S69. 10.1016/j.jfma.2023.03.022 PMC10101546 37061399

[15] Papathanasiou J , Petrov I , Kashilska Y , Kostov K , Dzhafer N . Is telerehabilitation a top priority for the Bulgarian healthcare system in the post COVID‐19 era? Health Policy Technol. 2022;11(4):100664. 36033103 10.1016/j.hlpt.2022.100664 PMC9398819

[16] Mavronasou A , Asimakos A , Vasilopoulos A , Katsaounou P , Kortianou EA . Remote administration of the short physical performance battery, the 1‐minute sit to stand, and the Chester step test in post‐COVID‐19 patients after hospitalization: establishing inter‐reliability and agreement with the face‐to‐face assessment. Disabil Rehabil. 2024;46:5334‐5344. 38156771 10.1080/09638288.2023.2297928

[17] Plaza M , de la Morales MB , de Sevilla GGP , et al. Telematics program of breathing exercises and mindfulness for post‐coronavirus disease 2019 patients. Rev Assoc Medica Bras. 1992;68(5):632‐635. 10.1590/1806-9282.20211336 35584487

[18] de la Plaza San Frutos M , Abuín Porras V , Blanco Morales M , et al. Telemedicine in pulmonary rehabilitation – benefits of a telerehabilitation program in post‐COVID‐19 patients: a controlled quasi‐experimental study. Ther Adv Respir Dis. 2023;17:17534666231167354. Available from: https://www.ncbi.nlm.nih.gov/pmc/articles/PMC10151922/ 37119059 10.1177/17534666231167354 PMC10151922

[19] Rodriguez‐Blanco C , Bernal‐Utrera C , Anarte‐Lazo E , Gonzalez‐Gerez JJ , Saavedra‐Hernandez M . A 14‐day therapeutic exercise telerehabilitation protocol of physiotherapy is effective in non‐hospitalized post‐COVID‐19 conditions: a randomized controlled trial. J Clin Med. 2023;12(3):776. 36769425 10.3390/jcm12030776 PMC9918076

[20] Calvache‐Mateo A , Heredia‐Ciuró A , Martín‐Núñez J , et al. Efficacy and safety of respiratory telerehabilitation in patients with long COVID‐19: a systematic review and meta‐analysis. Healthcare. 2023;11(18):2519. 37761716 10.3390/healthcare11182519 PMC10530340

[21] Calvo‐Paniagua J , Díaz‐Arribas MJ , Valera‐Calero JA , et al. A tele‐health primary care rehabilitation program improves self‐perceived exertion in COVID‐19 survivors experiencing post‐COVID fatigue and dyspnea: a quasi‐experimental study. PLoS One. 2022;17(8):e0271802. 35926004 10.1371/journal.pone.0271802 PMC9352012

[22] Kortianou EΑ , Tsimouris D , Mavronasou A , et al. Application of a home‐based exercise program combined with tele‐rehabilitation in previously hospitalized patients with COVID‐19: a feasibility single‐cohort interventional study. Pneumonologie. 2022;35(2):1‐10.

[23] Mheiri AA , Girish S , Amaravadi SK . Effects of six weeks of supervised telerehabilitation on pulmonary function, functional capacity, and Dyspnoea among individuals with long COVID. medRxiv. 2023. doi:10.1101/2023.09.27.23296254v1

[24] da Silva MMC , Viana DR , Colucci MG , et al. Effects of a cardiopulmonary telerehabilitation using functional exercises in individuals after COVID‐19 hospital discharge: a randomized controlled trial. J Telemed Telecare. 2023;31:311‐319. 37559399 10.1177/1357633X231188394

[25] del Corral T , Fabero‐Garrido R , Plaza‐Manzano G , Fernández‐de‐las‐Peñas C , Navarro‐Santana M , López‐de‐Uralde‐Villanueva I . Home‐based respiratory muscle training on quality of life and exercise tolerance in long‐term post‐COVID‐19: randomized controlled trial. Ann Phys Rehabil Med. 2023;66(1):101709. 36191860 10.1016/j.rehab.2022.101709 PMC9708524

[26] de Araújo Furtado PL , do Socorro Brasileiro‐Santos M , de Mello BLC , et al. The effect of telerehabilitation on physical fitness and depression/anxiety in post‐COVID‐19 patients: a randomized controlled trial. Int J Telerehabil. 2023;15(1):e6560. doi:10.5195/ijt.2023.6560 38046546 PMC10687992

[27] Dalbosco‐Salas M , Torres‐Castro R , Rojas Leyton A , et al. Effectiveness of a primary care telerehabilitation program for post‐COVID‐19 patients: a feasibility study. J Clin Med. 2021;10(19):4428. 34640447 10.3390/jcm10194428 PMC8509356

[28] McDonagh ST , Dalal H , Moore S , et al. Home‐based versus centre‐based cardiac rehabilitation. Cochrane Heart Group editor. Cochrane Database Syst Rev. 2023;2023(10):CD007130. doi:10.1002/14651858.CD007130.pub5 PMC10604509 37888805

[29] Vanzella LM , Cotie LM , Flores‐Hukom M , Marzolini S , Konidis R , Ghisi GLDM . Patients' perceptions of hybrid and virtual‐only care models during the cardiac rehabilitation patient journey: a qualitative study. J Cardiovasc Nurs. 2024;40:E91‐E100. doi:10.1097/JCN.0000000000001076 38206327

[30] Teixeira Do Amaral V , Viana AA , Heubel AD , et al. Cardiovascular, respiratory, and functional effects of home‐based exercise training after COVID‐19 hospitalization. Med Sci Sports Exerc. 2022;54(11):1795‐1803. 35714077 10.1249/MSS.0000000000002977

[31] Philip KEJ , Owles H , McVey S , et al. An online breathing and wellbeing programme (ENO breathe) for people with persistent symptoms following COVID‐19: a parallel‐group single‐blind randomised controlled trial. Lancet Respir Med. 2022;10(9):851‐862. 35489367 10.1016/S2213-2600(22)00125-4 PMC9045747

[32] Duncan PW , Bernhardt J . Telerehabilitation: has its time come? Stroke. 2021;52(8):2694‐2696. 34192896 10.1161/STROKEAHA.121.033289

[33] López C , Closa C , Lucas E . Telemedicina en rehabilitación: necesidad y oportunidad post‐COVID. Rehabilitación. 2020;54(4):225‐227. 32736803 10.1016/j.rh.2020.06.003 PMC7345413

[34] Antoniou KM , Vasarmidi E , Russell AM , et al. European Respiratory Society statement on long COVID follow‐up. Eur Respir J. 2022;60(2):2102174. 35144991 10.1183/13993003.02174-2021 PMC9349784

[35] Li J , Xia W , Zhan C , et al. A telerehabilitation programme in post‐discharge COVID‐19 patients (TERECO): a randomised controlled trial. Thorax. 2022;77(7):697‐706. 34312316 10.1136/thoraxjnl-2021-217382 PMC8318721

[36] Udina C , Ars J , Morandi A , Vilaró J , Cáceres C , Inzitari M . Rehabilitation in adult post‐COVID‐19 patients in post‐acute care with therapeutic exercise. J Frailty Aging. 2021;10(3):297‐300. 34105716 10.14283/jfa.2021.1 PMC7876526

[37] Mohamed AA , Alawna M . The effect of aerobic exercise on immune biomarkers and symptom severity and progression in patients with COVID‐19: a randomized control trial. J Bodyw Mov Ther. 2021;28:425‐432. 34776174 10.1016/j.jbmt.2021.07.012 PMC8339452

[38] Gonzalez‐Gerez JJ , Saavedra‐Hernandez M , Anarte‐Lazo E , Bernal‐Utrera C , Perez‐Ale M , Rodriguez‐Blanco C . Short‐term effects of a respiratory telerehabilitation program in confined COVID‐19 patients in the acute phase: a pilot study. Int J Environ Res Public Health. 2021;18(14):7511. 34299962 10.3390/ijerph18147511 PMC8306449

[39] Rodriguez‐Blanco C , Gonzalez‐Gerez JJ , Bernal‐Utrera C , Anarte‐Lazo E , Perez‐Ale M , Saavedra‐Hernandez M . Short‐term effects of a conditioning telerehabilitation program in confined patients affected by COVID‐19 in the acute phase. A pilot randomized controlled trial. Medicina Mex. 2021;57(7):684. 10.3390/medicina57070684 PMC8305888 34356965

[40] Pinto M , Gimigliano F , De Simone S , Costa M , Bianchi AAM , Iolascon G . Post‐acute COVID‐19 rehabilitation network proposal: from intensive to extensive and home‐based IT supported services. Int J Environ Res Public Health. 2020;17(24):9335. 33327384 10.3390/ijerph17249335 PMC7764833

[41] Carpes MF , Mayer AF , Simon KM , Jardim JR , Garrod R . The Brazilian Portuguese version of the London chest activity of daily living scale for use in patients with chronic obstructive pulmonary disease. J Bras Pneumol. 2008;34(3):143‐151. 18392462 10.1590/s1806-37132008000300004

[42] Silva IJCS , Barbosa GB , Isoppo KDS , Karloh M , Mayer AF . Reliability and validity of the online application of London chest activity of daily living scale in assessing dyspnea‐related functional impairment in individuals after hospitalization for COVID‐19. Disabil Rehabil. 2024;46:5618‐5623. 38226600 10.1080/09638288.2024.2303366

[43] Tanhan A , Ozer AY , Timurtas E , Batirel A , Polat MG . Is asynchronous telerehabilitation equal to synchronous telerehabilitation in COVID‐19 survivors with classes 4–6? J Telemed Telecare. 2023;31:347‐358. 37545432 10.1177/1357633X231189761

[44] Castro APCR , Nascimento J d S , Palladini MC , Pelloso LRC d A , Barbosa MHL . Dor no Paciente com Síndrome Pós‐COVID‐19. Rev Cienc Hosp St Isabel. 2021;5(2):56‐62.

[45] Oliveira FR . Um novo sistema e‐Health para monitoramento remoto de pacientes em atenção domiciliar. A novel e‐Health system for remote monitoring of patients at home care. 2023 Mar 30. Available from: https://repositorio.ufu.br/handle/123456789/37907

[46] Lima CSP da C , Winkler I , Senna de V . Sobreviventes do Covid e do AVC têm em comum uma jornada de reabilitação: reflexões sobre a afasia e tecnologia. Cuad Educ Desarro. 2023;15(1):434‐451.

[47] da Mata AA , Silva ACFL e , Bernardes F d S , et al. Impacto da pandemia de COVID‐19 na saúde mental de crianças e adolescentes: uma revisão integrativa/the impact of COVID‐19 pandemic on mental health of children and adolescents: an integrative review. Braz J Dev. 2021;7(1):6901‐6917. doi:10.34117/bjdv7n1-466

[48] Wang G , Zhang Y , Zhao J , Zhang J , Jiang F . Mitigate the effects of home confinement on children during the COVID‐19 outbreak. Lancet Lond Engl. 2020;395(10228):945‐947. 10.1016/S0140-6736(20)30547-X PMC7124694 32145186

[49] Brooks SK , Webster RK , Smith LE , et al. The psychological impact of quarantine and how to reduce it: rapid review of the evidence. Lancet. 2020;395(10227):912‐920. 32112714 10.1016/S0140-6736(20)30460-8 PMC7158942

[50] Loades ME , Chatburn E , Higson‐Sweeney N , et al. Rapid systematic review: the impact of social isolation and loneliness on the mental health of children and adolescents in the context of COVID‐19. J Am Acad Child Adolesc Psychiatry. 2020;59(11):1218‐1239.e3. 32504808 10.1016/j.jaac.2020.05.009 PMC7267797

[51] Pereira PSP d F . Impacto de programas de reabilitação respiratória na função respiratória de doentes COVID‐19 em fase pós‐aguda: uma revisão sistemática da literatura. 2021 Available from: http://hdl.handle.net/10198/23767

[52] Liu K , Zhang W , Yang Y , Zhang J , Li Y , Chen Y . Respiratory rehabilitation in elderly patients with COVID‐19: a randomized controlled study. Complement Ther Clin Pract. 2020;39:101166. 32379637 10.1016/j.ctcp.2020.101166 PMC7118596

[53] Gimigliano F , Young VM , Arienti C , et al. The effectiveness of behavioral interventions in adults with post‐traumatic stress disorder during clinical rehabilitation: a rapid review. Int J Environ Res Public Health. 2022;19(12):7514. Available from: https://www.ncbi.nlm.nih.gov/pmc/articles/PMC9224304/ 35742762 10.3390/ijerph19127514 PMC9224304

[54] Ju N , Yang X , Ma X , et al. Hospitalization, interpersonal, and personal factors of social anxiety among COVID‐19 survivors at the six‐month follow‐up after hospital treatment: the minority stress model. Eur J Psychotraumatol. 2022;13(1):2019980. doi:10.1080/20008198.2021.2019980 35111284 PMC8803063

[55] Gonçalves JVRD . Saúde mental e telessaúde: o potencial de redução de custos dos reinternamentos. 2022 Available from: https://run.unl.pt/handle/10362/154216

[56] Oncu F . Virtualization of mental health care in the midst of chaos: is telepsychiatry a silver lining? J Psychiatry Neurol Sci. 2021;34:219‐222.

[57] Nacarato D . Telerreabilitação cardiovascular: capacidade funcional, aptidão cardiorrespiratória e qualidade de vida em idosos: revisão sistemática e meta‐análise. 2022 Available from: https://www.repositorio.unicamp.br/acervo/detalhe/1262332

[58] OECD . Health at a Glance 2021: OECD Indicators. Organisation for Economic Co‐operation and Development; 2021 Available from: https://www.oecd‐ilibrary.org/social‐issues‐migration‐health/health‐at‐a‐glance‐2021_ae3016b9‐en

[59] Leochico CFD , Espiritu AI , Ignacio SD , Mojica JAP . Challenges to the emergence of telerehabilitation in a developing country: a systematic review. Front Neurol. 2020;11:1007. 33013666 10.3389/fneur.2020.01007 PMC7505991

[60] Baffert S , Hadouiri N , Fabron C , Burgy F , Cassany A , Kemoun G . Economic evaluation of telerehabilitation: systematic literature review of cost‐utility studies. JMIR Rehabil Assist Technol. 2023;10:e47172. 37669089 10.2196/47172 PMC10509745

[61] Seelman KD , Hartman LM . Telerehabilitation: policy issues and research tools. Int J Telerehabil. 2009;1(1):47‐58. 25945162 10.5195/ijt.2009.6013 PMC4296776

[62] Roine R , Ohinmaa A , Hailey D . Assessing telemedicine: a systematic review of the literature. CMAJ. 2001;165(6):765‐771. 11584564 PMC81454

[63] Ribeiro AM , Ribeiro BP , Petry LS , Dias LR d S , Heberle SM , Fagundes SL . Relatos de experiências acadêmicas do curso de fisioterapia em projeto de reabilitação e telerreabilitação de pacientes pós‐alta da COVID‐19. An Most Iniciaç Científica CESUCA. 2015;2(3): Available from: https://ojs.cesuca.edu.br/index.php/mostrac/article/view/2152

