# Supplementary figures and images for: Telerehabilitation as an innovative strategy for the management of anxiety and dyspnea in post‐COVID‐19: A scoping review
Source: PM R. 2025 May 27;17(11):1366–80. doi: 10.1002/pmrj.13403 (PMC12632175; doi:10.1002/pmrj.13403)

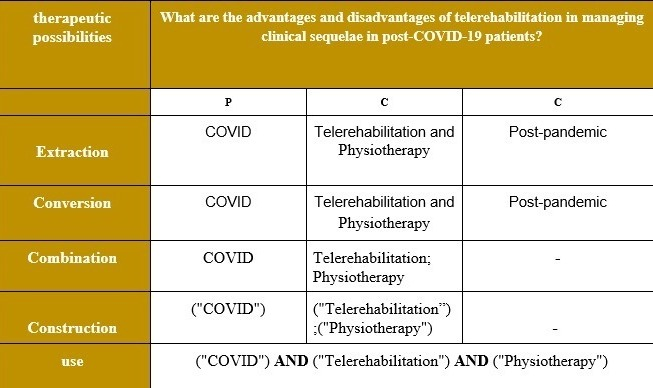

Supplement: Supplementary file 1 — Table S1 [file PMRJ-17-1366-s001.tiff]
